# Assessing wildfire extents in Siberian forests using machine learning

**DOI:** 10.1038/s41598-025-17465-5

**Published:** 2025-09-25

**Authors:** Ivan P. Malashin, Igor Masich, Vladimir Nelyub, Aleksei Borodulin, Andrei Gantimurov, Vadim Tynchenko

**Affiliations:** Bauman Moscow State Technical University, 105005 Moscow, Russia

**Keywords:** Wildfire prediction, Fire size estimation, Machine learning, Siberian forests, Climate factors, Climate sciences, Environmental sciences, Natural hazards

## Abstract

Wildfires significantly impact ecosystem dynamics and forest management strategies globally, including in Siberian forests. This study develops a machine learning (ML) framework to estimate wildfire size by integrating meteorological variables, forest composition, detection techniques, and historical fire records within the Krasnoyarsk Krai region of central Siberia. The dataset includes temperature, humidity, wind speed, precipitation, geospatial coordinates, and proximity to human settlements, which are used to train multiple predictive models, including XGBoost, Random Forest, K-Nearest Neighbors, Logistic Regression, and Decision Tree. XGBoost achieved the highest classification accuracy of 88.8%, outperforming other methods. Feature importance analysis highlights the influence of urban proximity, wind patterns, and meteorological conditions related to fuel moisture on fire size prediction. SHAP (SHapley Additive exPlanations) analysis indicates that smaller fires are associated with localized weather conditions, while extended dry periods correspond to larger fire events. While these results demonstrate the potential of ML for fire size classification in this specific region, the framework should be considered exploratory and region-specific. Future applications to other areas will require local data calibration.

## Introduction

Forest fires in Siberian ecosystems significantly impact forest structure and function, disrupting climate regulation, carbon storage, and biodiversity. In the Krasnoyarsk Territory, expansive boreal forests, coupled with unique climatic conditions, contribute to a complex fire regime that affects soil quality, air purity, and regional economies. Wildfires in this region result from a combination of meteorological factors, forest composition, and human influence, creating substantial challenges for effective forest management. Fire has been an inherent component of forest dynamics throughout history, shaping forest formation, distribution, productivity, and ecological processes. It remains a key factor influencing the state and evolution of forests, with profound effects on both ecological health and resource potential. This issue is shared globally, with countries such as the USA, Canada, Brazil, Spain, Portugal, France, and Australia also grappling with high forest flammability. While anthropogenic factors are the primary drivers of forest fires, their consequences can be both destructive and regenerative, ranging from forest degradation to ecological recovery.

Recent advances in data analysis, including the application of machine learning (ML) techniques, offer new ways to explore the complex relationships between climate variables, forest characteristics, and fire behavior. While traditional statistical models have been used to identify general patterns in wildfire occurrence, the application of ML for fire size assessment in Siberian forests has not been thoroughly examined.

Coffield et al.^1^ conducted a study in Alaska focusing on a 17-year period from 2001 to 2017, analyzing wildfire data from the Alaska Large Fire Database (ALFD) and MODIS satellite fire data. The dataset was filtered to include only fires in the ‘limited’ fire management zone, where minimal suppression occurs. Meteorological data, such as temperature, relative humidity, and vapor pressure deficit (VPD), were obtained from the ERA5 reanalysis, while vegetation data from LANDFIRE and topographical information from the USGS DEM were also incorporated. A decision tree (DT) ^2^ model was developed to predict final fire size based on these factors. The model aimed to capture fire dynamics, interannual variability, and assess the influence of human fire management. The limitations of the study include the use of fire data only from the zone with minimal fire suppression, which restricts the ability to generalize to other zones with more active suppression efforts ^3^. Additionally, the reliance on satellite and meteorological data, which may have spatial and temporal discrepancies, poses another limitation.

O et al.^4^ focused on fire perimeter prediction in a 34,000 $\text {km}^{2}$ area spanning the northern Rocky Mountains across Idaho, Nevada, and Utah, incorporating a variety of vegetation types. The research involved 238 fires with complete perimeters, recorded from 1984 to 2014. To model fire perimeter locations, the study used predictor variables such as elevation, slope, fuel type, and distance from roads and rivers, and incorporated compound fire indices like resistance to control and suppression difficulty. Boosted regression tree (BRT) model ^5^ was applied to assess spatial relationships between fire occurrence and landscape features, while cross-validation and AUC metrics evaluated model performance. Results highlighted the influence of barrier distance, travel cost, suppression difficulty index, and valley proximity on fire perimeter likelihood. The model showed a 69% prediction accuracy and was used to identify high and low likelihood fire perimeter locations across the landscape, providing a tool for future fire control efforts. The study faces a few challenges, including the potential inaccuracy of data like fire perimeter mapping and limited information about the impact of past fires. Additionally, the model relies on ideal weather conditions for predictions, which may not always align with actual conditions ^6^.

Pu Mat National Park in Nghe An Province, Vietnam, covering approximately 94,804 ha and characterized by a tropical monsoon climate with altitudinal variation in temperature and a five-month drought period. An inventory map of 56 historical fire perimeters from 2014 to 2016 was developed using georeferenced records verified by field surveys. Nine explanatory variables, including topography, climate (annual temperature and drought index), hydrology (river density), land cover, and proximity to roads and residential areas, were compiled and converted into a 30 $\times$ 30 m raster format. The Relief-F feature selection method was employed by Pham et al. ^7^ to rank the influence of these variables on fire occurrence, revealing that human-related factors were significant. BRT approach was then used to model fire perimeter presence by integrating these predictor variables, with model performance evaluated using cross-validation and AUC metrics. The resulting model produced a continuous probability surface of fire perimeter likelihood, providing insights into areas that may require targeted fire suppression and mitigation efforts.

Predicting the spread of forest fires accurately is important for effective risk management and firefighting efforts. Sun et al.^8^ introduces the Forest Fire Spread Behavior Prediction (FFSBP) model, which forecasts the fire’s direction and speed using a combination of Cellular Automata (CA) ^9^ and the Wang Zhengfei model ^10^. The FFSRP model, on the other hand, predicts the extent of the burned area using ML methods. The effectiveness of the models is validated through case studies, such as the “3.29 Forest Fire” in China ^11^, with results showing smaller relative errors compared to other simulation models. These findings highlight the potential of the FFSBP model to enhance fire prediction accuracy and support better risk mitigation strategies.

Forest fires in tropical seasonal forests significantly impact ecosystems, contributing to biodiversity loss and climate change. Addressing forest fires in these regions is critical for ecosystem conservation and effective management strategies. Mishra et al.^12^ analyzes forest fires trends, patterns, and distribution from 2001 to 2022 in Odisha, India, utilizing MODIS imagery, Kernel density tools, and Support Vector Machine (SVM) ^13^ and Random Forest (RF) ^14^ to predict forest fire risk. The findings reveal that 20.14% and 16.72% of Odisha’s area are highly susceptible to FF, with the highest fire occurrences in Angul district. The upward trend in fire incidents, especially post-2015, emphasizes the urgency for region-specific management strategies. Insights from global forest fires management practices help inform local prevention and mitigation strategies.

Sharme at al.^15^ investigates forest fire prediction in South Carolina (SC) using ML and neural network techniques. Data from the SC Forestry Commission for 2023 were utilized, incorporating meteorological, terrain, vegetation, and infrastructure factors. Various models, including DT, RF, Logistic Regression (LR), SVM, and Artificial Neural Network (ANN) ^16^, were evaluated. The DT model achieved the highest accuracy of 90.58%, but the correlation test-based method generated a more accurate fire hazard map. The study highlighted the importance of correlation coefficients over feature importance in predicting fire hazards. The overlap of fire-prone areas and carbon hotspots highlight the need for targeted efforts to mitigate carbon loss and climate change. These findings contribute to improving fire prediction and prevention in SC.

Forest fires disrupt biodiversity, air quality, and local economies, posing severe challenges in the context of climate change. Do et al.^17^ focuse on predicting fire risks in Dak Nong, a highland province in Vietnam where forest farming is prevalent. UAV-based remote sensing data revealed a decline in forest cover alongside an increase in agricultural rubber and industrial crops. The integration of SPOT satellite images with UAV data and the XGBoost algorithm achieved high accuracy (OA = 81.446%, Kappa = 0.803) in mapping forest types. A hybrid model combining Artificial Bee Colony and Adaptive Neuro Fuzzy Inference System (ABC-ANFIS) was used to assess fire susceptibility, indicating that most forests, especially bamboo and dipterocarp areas, are at high to very high risk of fire. These findings provide valuable insights for developing improved fire prevention and suppression strategies that can be adapted to similar regions.

Although there is a vast body of studies on wildfire prediction and modeling, research on estimating fire size in Siberian forests, specifically in the Krasnoyarsk region, remains limited. ML methods have not been applied to this issue in this area. Fire size can be influenced by factors such as climate conditions, forest composition, and topographical features. Research focusing on these variables could help develop models to estimate fire size based on these factors, supporting forest management practices and inform strategies for wildfire risk mitigation.

The paper describes the processes of data collection and preprocessing, the selection and justification of ML algorithms–namely XGBoost, Random Forest, K-Nearest Neighbors, Logistic Regression, Decision Trees. Emphasis is placed on how variations in climatic conditions and forest characteristics relate to fire sizes, with the goal of providing actionable insights for forest managers and policymakers working in Siberian regions.

## Materials and methods

### Study area

**Figure 1:**
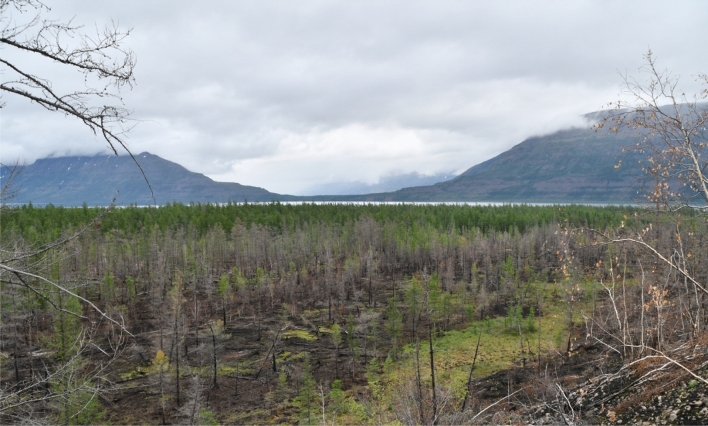
Burned forest in the northern taiga near Lake Lama on the Putorana Plateau, Krasnoyarsk Krai.

Krasnoyarsk Krai is a vast and ecologically diverse region located in Siberia, Russia. It spans over 2.3 million square kilometers, making it one of the largest administrative divisions in the country. The region’s landscape is characterized by a mix of taiga forests, mountain ranges, river valleys, and vast plains. The Krai is home to a variety of ecosystems, from boreal forests to tundra, depending on the geographical area. The forested regions of Krasnoyarsk Krai are prominent, with coniferous forests (mainly pine, spruce, and fir) dominating the central and northern parts, while mixed forests, including deciduous trees such as birch and aspen, are found in the southern regions. These forests regulate the local climate, maintaining biodiversity, and supporting the livelihoods of local communities. Figure 1 shows the aftermath of a wildfire, highlighting the impact of such fires on the region’s coniferous forests.

The forests of Krasnoyarsk Krai are a key part of the Russian forest zone, one of the largest forested areas in the world. These ecosystems are rich in biodiversity and are vital for carbon sequestration, acting as natural climate regulators. However, they also face threats from wildfires, especially in the summer months, when dry conditions, heatwaves, and strong winds create an ideal environment for fire spread. The region’s fire-prone forests, coupled with human activities and climatic factors, contribute to the high frequency and intensity of wildfires.

**Figure 2:**
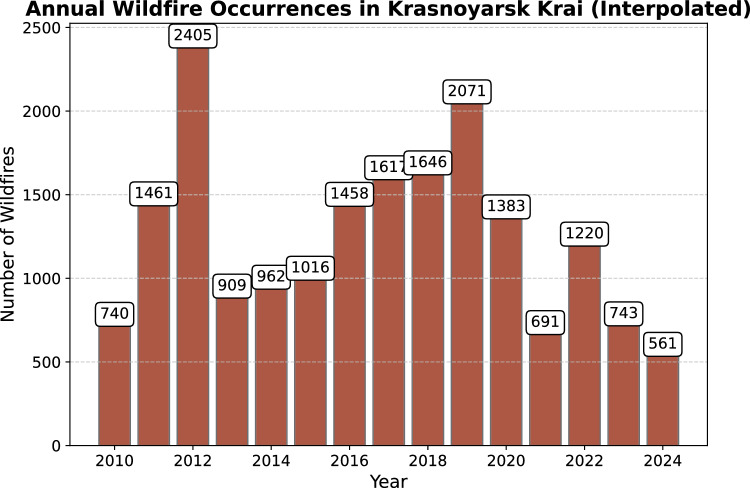
Annual wildfire occurrences in Krasnoyarsk Krai.

Figure 2 shows annual wildfire distribution from 2010 to 2024 years. In years with higher wildfire counts–such as 2012, 2019, 2018, and 2017 – extended dry periods, high summer temperatures, and low precipitation contributed to drier vegetation, making forests more susceptible to ignition. Additionally, increased lightning activity during thunderstorms and higher levels of human-caused ignition events could have further elevated fire numbers in these years.

Conversely, lower wildfire counts in years like 2024, 2021, and 2023 may indicate periods with more favorable moisture conditions. Increased precipitation, lower temperatures, or shorter fire seasons would result in higher vegetation moisture and reduced fuel availability, thereby decreasing the likelihood of fires ^18^. Effective fire management and prevention strategies implemented during these periods might also have contributed to the reduction in fire occurrences ^19^.

**Figure 3:**
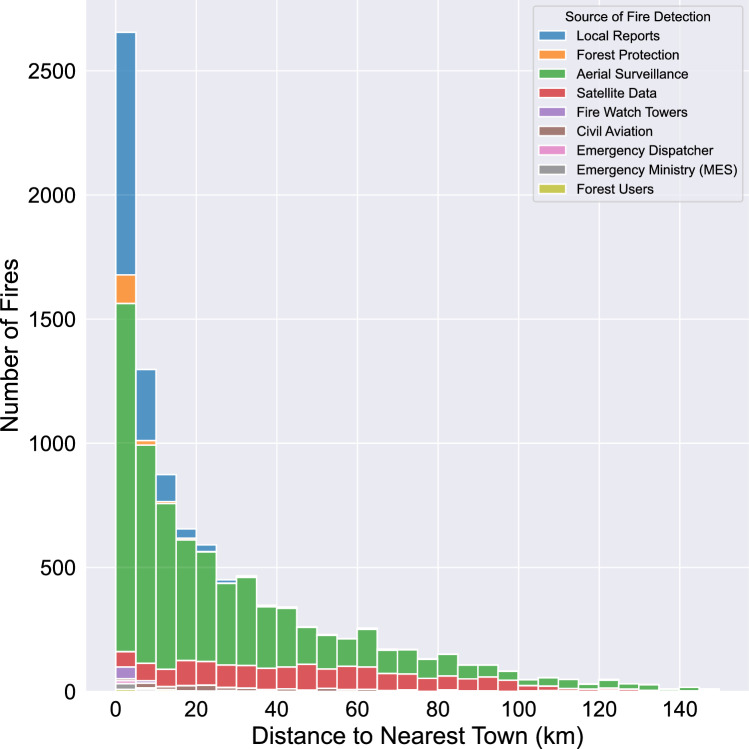
Annual wildfire occurrences in Krasnoyarsk Krai.

Figure 3 shows the distribution of wildfire occurrences based on the distance to the nearest town, categorized by detection method. The histogram represents the number of fires detected within 150 km of a town, with colors indicating different detection sources. Data smoothing was applied to remove outliers. The most common detection methods include satellite observations, ground patrols, and aerial surveillance.

### Fire size statistics

**Figure 4:**
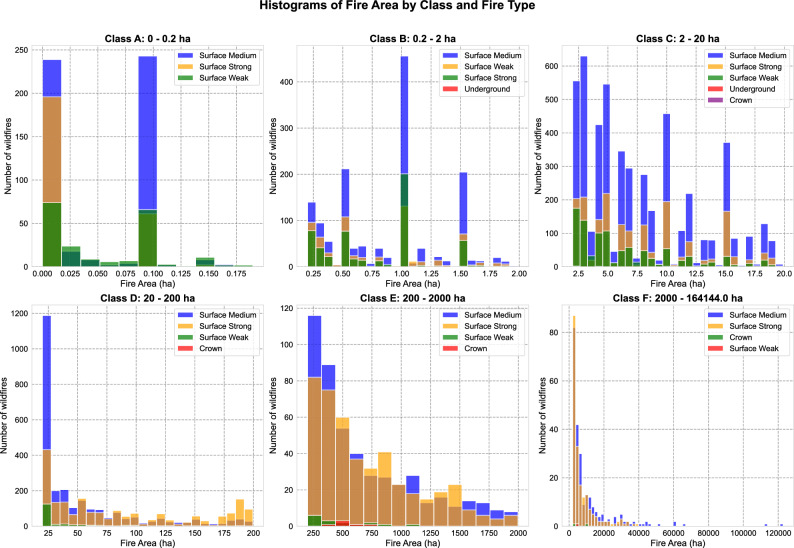
Distribution of wildfire areas in Krasnoyarsk Krai across six fire size classes (A–F), stratified by fire type. Each panel represents a distinct size category ranging from very small fires (Class A: < 0.2 ha) to extreme fires (Class F: >2000 ha), with fire types including surface fires (weak, medium, strong), underground fires, and crown fires. The histograms reveal shifting fire-type compositions and frequency distributions as fire size increases–from predominantly weak surface fires in smaller classes to a higher prevalence of crown and strong surface fires in larger classes.

Wildfire classification by burn area helps assess severity and response needs. Class A ($\le$0.2 ha) includes small ignitions that are easily controlled. Class B (0.2–2 ha) covers minor fires with limited impact. Class C (2–20 ha) consists of moderate fires requiring firefighting efforts. Class D (20–200 ha) represents large fires that are difficult to contain. Class E (200–2000 ha) includes major wildfires causing damage. Class F (>2000 ha) refers to catastrophic fires ^20^ with widespread destruction. Higher classes demand more resources and pose greater environmental and societal risks.

The distribution of wildfire sizes (Fig. 4) in Krasnoyarsk Krai from 2010 to 2024 reveals distinct patterns influenced by climate, vegetation, and fire behavior dynamics. The majority of fires fall into the small-scale categories (Classes A and B, 0–2 ha), dominated by weak-to-medium surface fires. These are often caused by localized ignition sources such as human activity (discarded cigarettes, agricultural burns, equipment sparks) or natural events like lightning strikes in dry conditions ^21^. Due to their limited fuel load and quick suppression efforts, they rarely escalate into larger fires. Some underground fires appear in Class B, in peatland areas, where smoldering fires can persist for long periods despite their small initial size.

As fire size increases, the intensity and type of fire change significantly. Moderate fires (Class C, 2–20 ha, and Class D, 20–200 ha) show a notable rise in strong surface and crown fires, suggesting that fires of this scale often occur in dense boreal forests where dry needles, fallen branches, and undergrowth provide extensive fuel. Crown fires, which involve the burning of tree canopies, become more frequent in these classes, indicating more extreme fire behavior driven by prolonged droughts, strong winds, and high temperatures ^22^. These fires spread rapidly, often affecting large areas before containment efforts can take place.

The largest fires, categorized as Class E (200–2000 ha) and Class F (>2000 ha, up to 164,144 ha), represent the most destructive wildfire events, primarily consisting of high-intensity surface and crown fires. These fires are relatively rare, with fewer than 100 occurrences over the 14-year period, yet they contribute disproportionately to total burned area. The driving factors behind these massive fires include prolonged summer droughts, strong winds that facilitate rapid fire spread, and the vast ^23^, continuous stretches of forest typical of central and eastern Krasnoyarsk Krai. Unlike smaller fires, which may burn for only a few hours or days, Class E and F fires can last for weeks or even months, often requiring large-scale firefighting operations.

**Figure 5:**
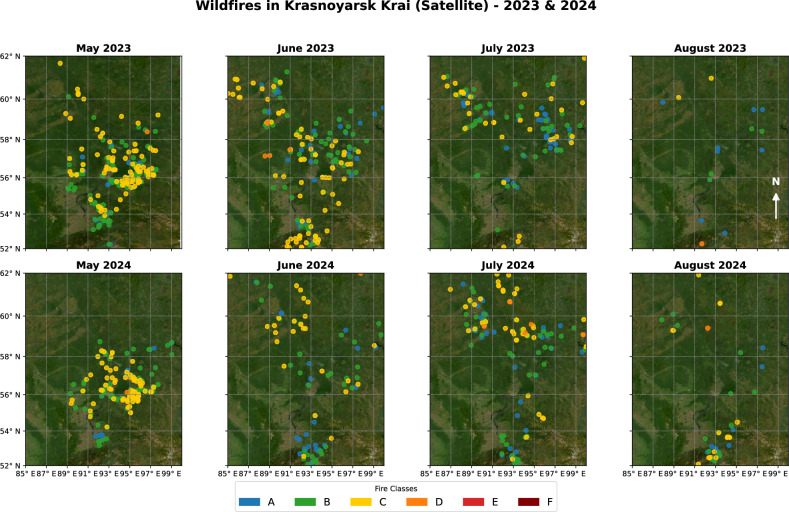
Spatial distribution of wildfires in central part of Krasnoyarsk Krai during May–August 2023 and 2024, with fires classified by size.

A key factor influencing the escalation of fires into these larger categories is fuel accumulation ^24^ In remote forested areas with limited fire suppression, decades of deadwood buildup, thick undergrowth, and dry organic material create conditions where a single ignition source can lead to an uncontrollable wildfire. Climate change exacerbates this risk by increasing the frequency of heatwaves and reducing precipitation, making forests drier for longer periods. Additionally, strong wind patterns play a crucial role in the spread of crown fires, allowing embers to ignite new fires kilometers away from the original source.

The fire size distribution highlights the growing threat of megafires ^25^ (Classes E and F) driven by climate extremes, while small-to-moderate fires (Classes A to D) remain frequent due to human activity and localized weather conditions. The increasing frequency of larger, more intense fires suggests that fire management strategies must adapt to account for longer fire seasons, reduced water availability, and the rising impact of climate change on Siberian forests.

Figure 5 shows the spatial distribution of wildfires in Krasnoyarsk Krai during 2023–2024, with clear clustering patterns that align with climatic gradients, dominant vegetation zones, and proximity to anthropogenic infrastructure. The highest concentration of fires is observed between $56^\circ$N and $60^\circ$N, corresponding to regions characterized by dense boreal forests (taiga) ^26^. These areas are dominated by coniferous species such as Siberian larch (Larix sibirica) ^27^, Scots pine (Pinus sylvestris) ^28^, and Siberian spruce (Picea obovata) ^29^, which are known for high flammability due to resinous litter and canopy structure. Additionally, fire density is elevated near transport corridors and logged areas, as seen through overlay analysis with road networks ^30^ and land use data (not shown), suggesting a combined effect of natural and anthropogenic factors. These forests provide a fuel load due to the accumulation of dry needles, fallen branches, and peat-rich soils, making them highly flammable, especially in dry conditions. The central and eastern parts of Krasnoyarsk Krai are vulnerable to large-scale wildfires (Class E and F fires), often driven by lightning strikes, prolonged summer droughts, and strong winds that facilitate rapid fire spread ^31,32^.

In contrast, northern areas (above $60^\circ$N) experience fewer fires due to colder temperatures, higher soil moisture, and the presence of permafrost. This region is characterized by sparse larch-dominated forests mixed with tundra landscapes, where wet and frozen ground limits fire propagation. When fires do occur, they are typically small (Class A and B) and slow-moving, affecting surface vegetation rather than the deep forest canopy. However, increasing temperatures in recent years may be reducing permafrost stability, potentially leading to more frequent fires in these areas in the future.

In the southern regions ($52-56^\circ$N), the landscape transitions from dense taiga forests to mixed and deciduous forests, featuring birch (Betula pendula), aspen (Populus tremula), and pine stands interspersed with grasslands ^33^. These areas experience more frequent but smaller fires (Class A, B, and C), often linked to human activities such as agriculture, logging, and settlement expansion. Due to higher population density, ignition sources such as discarded cigarettes, uncontrolled burning, and equipment sparks highly impact in fire outbreaks. Unlike the north, where fires are primarily natural, southern fires are often anthropogenic.

Seasonally, fires follow a latitudinal shift, starting in May–June in the south, where early-season dryness increases flammability, before spreading northward by July–August, when peak summer heat reduces humidity and dries out vegetation. The largest fires occur in mid-to-late summer in the central and eastern boreal forests, where drought, high temperatures, and low precipitation create ideal fire conditions ^34^.

The mapping results highlight the connection between climate, vegetation type, and fire risk. The central taiga forests are most prone to large-scale fires due to their high biomass and susceptibility to drought, while northern tundra and permafrost regions remain largely fire-resistant but could become more vulnerable with climate change. Meanwhile, southern forests and grasslands experience more frequent human-caused fires, though they are generally smaller in size. Given the increasing trend of warmer summers and drier conditions, wildfire risk in Krasnoyarsk Krai is expected to rise, in mid-latitude forested areas where fire suppression remains a challenge due to vast, inaccessible terrain ^35^.

### Climatic factors

**Figure 6:**
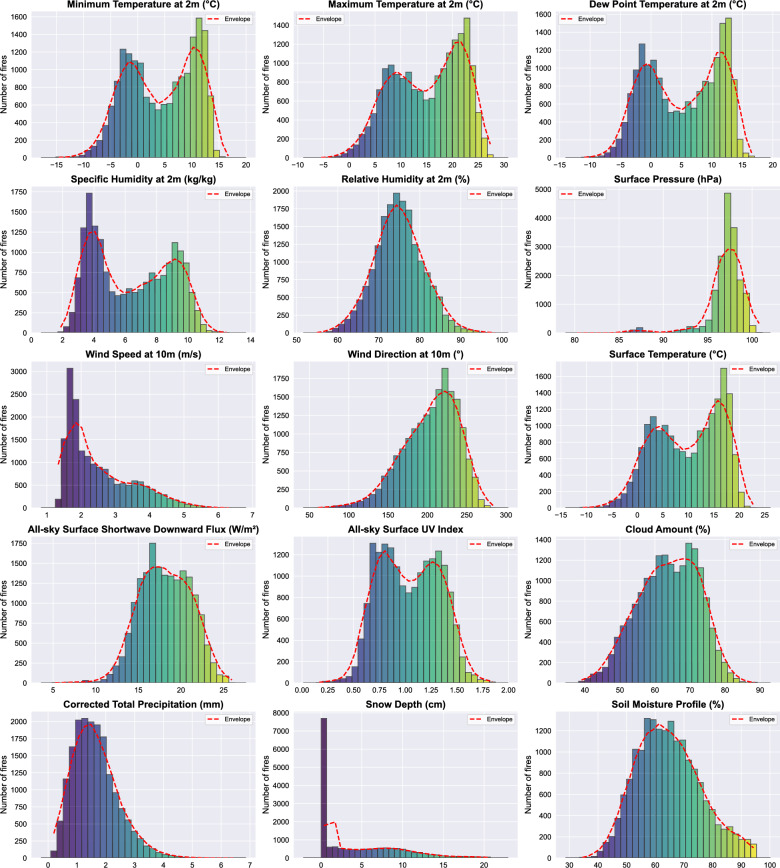
Histograms of averaged climatic conditions during the month preceding wildfire ignition events in Krasnoyarsk Krai (2010–2024). Each panel shows the distribution of a specific environmental variable–such as minimum/maximum temperature, dew point, humidity (specific and relative), wind speed and direction, surface pressure, soil moisture, cloud cover, UV index, solar radiation, precipitation, snow depth, and surface temperature–averaged over the 30 days prior to each recorded fire. These distributions illustrate the typical pre-fire atmospheric and surface conditions.

The analysis of climate conditions (Fig. 6) prior one month to wildfires in Krasnoyarsk Krai (2010–2024) reveals trends and correlations between meteorological variables and fire occurrences. Data aggregated over the month preceding each of the 17,921 fires in the region highlight key environmental factors contributing to fire risk. The minimum temperature at 2 meters varied between $-15^\circ$C and $+20^\circ$C, while the maximum temperature ranged from $-10^\circ$C to $+30^\circ$C. Fires were most frequent when minimum temperatures were around $5-15^\circ$C and maximum temperatures reached $20-25^\circ$C. Dew point temperatures ranged from $-15^\circ$C to $15^\circ$C, with peak fire occurrences corresponding to $5-10^\circ$C ^36^. Specific humidity values showed that fires were more frequent when humidity was between 5 and 10 g/kg. Relative humidity varied from 50% to 100%, with the highest number of fires occurring at around 60–70%. Fires were most common when wind speeds at 10m ranged from 2–5 m/s, indicating that moderate winds may facilitate fire spread ^37^. Surface pressure varied between 80–100 kPa, with a noticeable clustering of fires around 90–95 kPa. Fires were more frequent when surface shortwave downward flux was between 10–25 $\text {W/m}^{2}$, suggesting a strong correlation between solar radiation and fire risk ^38^. Cloud cover ranged from 40% to 90%, with fire numbers peaking when cloud cover was around 50–60%. Corrected total precipitation in the month before fires was typically low, often below 5 mm, while snow depth was minimal, usually under 20 cm. This indicates that fires were more likely in drier periods ^39^. Soil moisture percentages ranged from 30% to 100%, with most fires occurring when soil moisture was below 50%, reinforcing the role of dry conditions in fire susceptibility ^40^. Higher temperatures, especially above $20^\circ$C, increase fuel dryness and enhance the likelihood of ignition ^18^. The strong correlation between maximum temperatures of $20-25^\circ$C and fire frequency aligns with the critical threshold at which vegetation loses moisture and becomes highly flammable ^41^. The fact that fires peak when relative humidity is between 60–70% suggests that extreme dryness is not always necessary for ignition ^42^. Moderate humidity levels may sustain flammability while preventing the full saturation of fuels. Specific humidity in the 5–10 g/kg range also aligns with conditions where fine fuels remain dry enough to burn despite some atmospheric moisture ^43^. Moderate winds (2–5 m/s) provide enough airflow to oxygenate fires and spread embers without extinguishing flames, which explains why fires are more frequent in these conditions. Higher wind speeds could lead to rapid fire spread, while very low wind speeds might suppress fire activity ^44^. Increased solar radiation (10–25 $\text {W/m}^{2}$) contributes to surface heating and drying of vegetation, which in turn elevates fire risk ^45^. Fires are more common under partly cloudy conditions (50–60%), likely because full cloud cover reduces solar heating, while completely clear conditions might allow too much moisture loss, making fuels less flammable. Snow depth under 20 cm suggests that fires predominantly occur in periods where snow has either melted or failed to accumulate significantly ^46^. Fires occur most frequently when soil moisture is below 50%, reinforcing the idea that drought stress on vegetation directly influence in fire susceptibility ^47^. Drier soils also contribute to water stress in plants, making them more prone to burning ^48^. The data clearly illustrate that wildfires in Krasnoyarsk Krai are strongly influenced by a combination of high temperatures (above $20^\circ$C), low precipitation (<5 mm), moderate winds (2–5 m/s), and dry soils (<50% moisture). These findings suggest that climate trends, warming temperatures and shifts in precipitation patterns, could exacerbate wildfire risks in the future. As temperatures continue to rise, the frequency and intensity of wildfires may increase, posing challenges for fire management and disaster prevention strategies. Understanding these climatic influences can help in developing targeted mitigation measures, such as improved early warning systems, forest management practices, and policies aimed at reducing fire-prone conditions. Future research should focus on long-term climate projections and their potential impacts on wildfire behavior to better prepare for and mitigate the risks associated with climate-driven fire activity in the region.

### Fire indices

**Figure 7:**
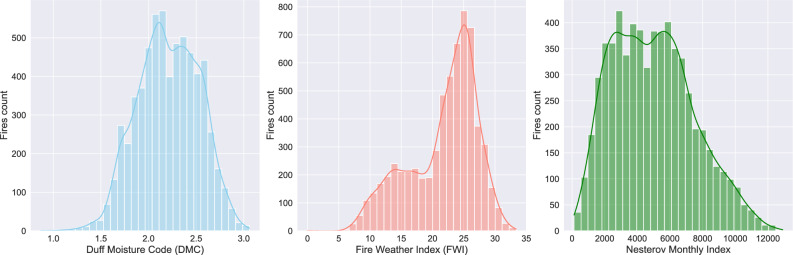
Fire indexes distribution.

Analysis of fire indices (Fig. 7) for locations where wildfires were recorded, based on climatic data from the month prior to each fire, reveals distinct trends in fire behavior and risk factors. The indices examined include the Duff Moisture Code ^49^ (DMC), the Fire Weather Index ^50^ (FWI), and the Nesterov Index ^51^, each quantifying fire risk based on different meteorological and fuel moisture conditions.

DMC represents the moisture content of decomposing organic layers in the forest floor, with higher values indicating drier conditions. It is calculated as:

1 $$\begin{aligned} \text {DMC} = DMC_{\text {previous}} + \left( \frac{0.92D}{1 + 0.0015DMC_{\text {previous}}} \right) \end{aligned}$$

where:

- $DMC_{\text {previous}}$ is the previous day’s DMC value,
- *D* is the daily temperature-dependent drying factor, which increases with temperature and decreases with precipitation.

Most wildfires occurred in areas where DMC values ranged from 1.0 to 3.0, suggesting that fires were more likely when the duff layer was relatively dry but not completely desiccated. These conditions favored smoldering combustion and fire spread through surface fuels.

FWI measures fire intensity potential and is derived from three sub-indices: the Fine Fuel Moisture Code (FFMC), DMC, and the Drought Code (DC). The FWI itself is calculated as:

2 $$\begin{aligned} \text {FWI} = 2.72 \times \left( \frac{ISI}{1 + e^{0.05039(DC - 80)}} \right) \end{aligned}$$

where:

- *ISI* (Initial Spread Index) is a function of wind speed and fine fuel moisture,
- *DC* (Drought Code) reflects long-term drying effects,
- *e* is the base of the natural logarithm.

Wildfires were concentrated in regions with FWI values between 5 and 35, indicating moderate-to-high fire danger conditions. Even at lower values (below 10), fires were still recorded, suggesting that ignition sources and fuel availability are impact, even when fire intensity was not extreme.

The Nesterov Index, used in Russia to assess fire hazard, is calculated as:

3 $$\begin{aligned} N = \sum (T_{\text {max}} - T_{\text {dew}}) P \end{aligned}$$

where:

- $T_{\text {max}}$ is the daily maximum temperature ($^\circ$C),
- $T_{\text {dew}}$ is the dew point temperature ($^\circ$C),
- *P* is a precipitation factor, set to zero if precipitation exceeds 3 mm, otherwise 1.

Wildfires were predominantly recorded in areas where Nesterov Index values ranged from 2,000 to 10,000, reflecting extended dry periods that increased vegetation flammability. This range suggests that while extreme dryness enhances fire likelihood, fires can also occur under moderate dryness if additional factors like wind or human activity contribute to ignition.

Fires occurred more frequently in areas with moderate-to-high DMC and FWI values, confirming that fuel dryness and atmospheric conditions influence wildfire development. Even when FWI was low, fires were recorded, indicating the importance of localized ignition sources and land-use activities in fire occurrences. The Nesterov Index data suggests that while prolonged dryness increases fire risk, moderate conditions can still lead to wildfires under the right conditions, such as human activity, wind, or fuel accumulation.

### Machine learning methods

A suite of ML algorithms was employed to model wildfire occurrence and classify fire sizes. XGBoost (Extreme Gradient Boosting) is an ensemble method based on gradient boosting decision trees, known for its high predictive accuracy, ability to capture nonlinear relationships, and built-in regularization ^52^. Random Forest, another ensemble-based method, constructs multiple decision trees using bootstrapped samples and random feature selection, providing strong performance in noisy data and reducing the risk of overfitting–a frequent concern in ecological modeling ^53^. K-Nearest Neighbors (KNN) is a non-parametric algorithm that classifies observations based on proximity in feature space, offering intuitive modeling of localized patterns in meteorological and spatial data ^54^, though it can be sensitive to data scaling and high dimensionality. Logistic Regression serves as a baseline linear classifier, useful for assessing the influence of individual predictors under the assumption of linearity ^55^. Decision Trees, while more prone to overfitting than their ensemble counterparts, provide interpretable hierarchical decision rules that are valuable for exploratory analysis and identifying key thresholds in fire-related variables ^56^.

To quantitatively assess fire size $S$ based on these monthly climatic data, one approach is to model $S$ using a multiple linear regression:

4 $$\begin{aligned} S = \alpha + \beta _1\,\overline{T}_{\min } + \beta _2\,\overline{T}_{\max } + \beta _3\,\overline{T}_{\text {dew}} + \beta _4\,\overline{H}_{\text {spec}} + \beta _5\,\overline{H}_{\text {rel}} + \beta _6\,\overline{W} + \beta _7\,\overline{P} + \beta _8\,\overline{I}_{\text {sw}} + \beta _9\,\overline{CC} + \beta _{10}\,\overline{SM} + \varepsilon , \end{aligned}$$

where:

- $\overline{T}_{\min }$ and $\overline{T}_{\max }$ denote the average minimum and maximum temperatures ($^\circ$C) over the month,
- $\overline{T}_{\text {dew}}$ is the average dew point temperature ($^\circ$C),
- $\overline{H}_{\text {spec}}$ and $\overline{H}_{\text {rel}}$ are the average specific humidity (g/kg) and relative humidity (%) respectively,
- $\overline{W}$ is the average wind speed (m/s),
- $\overline{P}$ represents precipitation (mm),
- $\overline{I}_{\text {sw}}$ is the average surface shortwave downward flux (W/m$^2$),
- $\overline{CC}$ is the average cloud cover (%),
- $\overline{SM}$ is the average soil moisture (%),
- $\alpha$ is the intercept, $\beta _i$ are the regression coefficients, and $\varepsilon$ is the error term.

Alternatively, a fire risk index (FRI) was constructed to encapsulate the combined effect of key climatic factors on fuel dryness and ignition potential:

5 $$\begin{aligned} FRI = \gamma \cdot \left( \frac{\overline{T}_{\max } - \overline{T}_{\min }}{\overline{P} + \delta } \right) \cdot \overline{W} \cdot \left( 1 - \frac{\overline{SM}}{100}\right) , \end{aligned}$$

where:

- $\gamma$ is a scaling constant,
- $\delta$ is a small constant to avoid division by zero,
- $\overline{T}_{\max } - \overline{T}_{\min }$ represents the diurnal temperature range,
- $\frac{1}{\overline{P} + \delta }$ reflects the impact of low precipitation on fuel dryness,
- $\overline{W}$ is the average wind speed (enhancing oxygen supply and ember dispersal),
- $1 - \frac{\overline{SM}}{100}$ quantifies the effect of reduced soil moisture.

For classification tasks (e.g., small vs. large fires), logistic regression can estimate the probability $P(\text {Large Fire})$:

6 $$\begin{aligned} P(\text {Large Fire}) = \frac{1}{1 + \exp \Bigl (-\Bigl (\theta _0 + \theta _1\,\overline{T}_{\max } + \theta _2\,\overline{H}_{\text {rel}} + \theta _3\,\overline{W} + \theta _4\,\overline{P} + \theta _5\,\overline{SM}\Bigr )\Bigr )}, \end{aligned}$$

where $\theta _0$ is the intercept and $\theta _i$ are the coefficients associated with the corresponding climatic factors.

For machine learning approaches, XGBoost was employed to predict fire size $S$, minimizing the following objective function:

7 $$\begin{aligned} \mathscr {L} = \sum _{i=1}^N L(S_i, \hat{S}_i) + \sum _{k=1}^K \Omega (f_k), \end{aligned}$$

where:

- $S_i$ denotes the observed fire size for the $i$-th sample,
- $\hat{S}_i$ is the predicted fire size,
- $L$ is a differentiable loss function (e.g., mean squared error),
- $\Omega (f_k)$ is a regularization term penalizing complexity,
- $N$ is the number of samples, and $K$ is the number of trees in the ensemble.

To interpret model predictions, SHAP (SHapley Additive exPlanations) analysis was conducted, decomposing predictions as:

8 $$\begin{aligned} f(x) = f_{\text {base}} + \sum _{i=1}^{n} \phi _i, \end{aligned}$$

where:

- $f_{\text {base}}$ is the mean model prediction over training data,
- $\phi _i$ represents the contribution of the $i$-th feature,
- $n$ is the number of features.

This framework, integrating statistical models and machine learning, enhances our understanding of wildfire dynamics, supporting targeted mitigation strategies.

### Proposed approach

This study develops a wildfire classification approach incorporating climatic conditions, forest structure, proximity to urban areas, and detection methods. The dataset combines multiple sources: atmospheric and surface parameters from the National Aeronautics and Space Administration (NASA) ^57^, wildfire occurrence records from the Krasnoyarsk Krai State Autonomous Institution “Lesopozharny Center” (KGAU) ^58^, and vegetation data from the regional Ministry of Natural Resources ^59^. Table 1 summarizes the key characteristics of each dataset, including spatial and temporal resolution, coverage period, and source access details

**Table 1:** Overview of datasets used for wildfire size classification in Krasnoyarsk Krai.

Source	Dataset name/type	Spatial resolution	Temporal resolution	Date range	Access/reference
NASA POWER Project	Atmospheric and surface meteorological variables	$\sim$ $0.5^\circ$ ( 55 km)	Daily	2010–2024	NASA POWER API ^57^
Krasnoyarsk Lesopozharny Center (KGAU)	Wildfire occurrence records	Point-based (fire locations)	Event-based (per fire)	2010–2024	KGAU website ^58^
Ministry of Natural Resources, Krasnoyarsk Krai	Forest composition and land cover	Variable (district-level)	Static / Periodic updates	Latest update: 2023	MLX Geoportal ^59^
OpenStreetMap / Regional GIS layers	Urban proximity and road networks	$\sim$10 m (vector data)	Static / Periodic updates	Latest update: 2024	OpenStreetMap / Regional datasets

**Figure 8:**
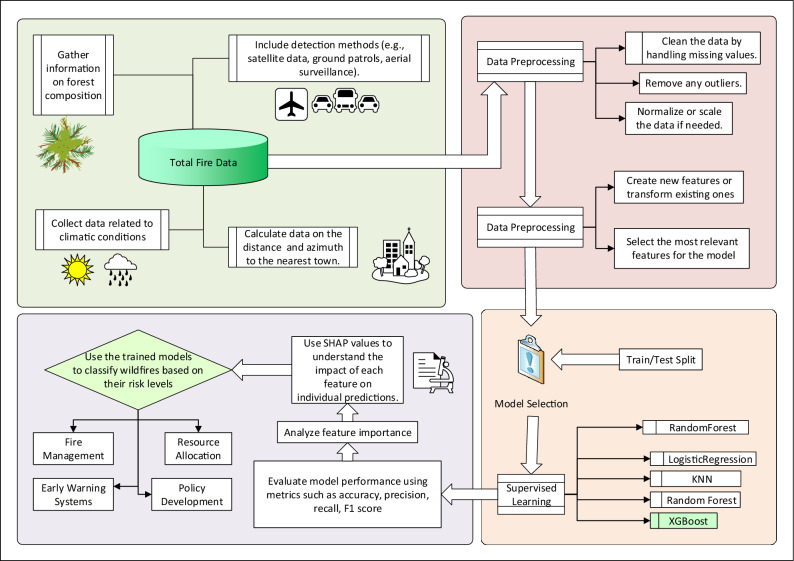
Workflow of the proposed approach.

The classification pipeline follows a structured workflow, progressing from data preprocessing to model interpretation, which shown in Fig. 8. To capture both local and landscape-level drivers of each fire event, meteorological and biophysical predictors were aggregated around the fire location at six nested spatial scales (A–F). Meteorological variables were sourced from the NASA POWER reanalysis at approximately $0.5^\circ$ ($\tilde{5}5$ km) grid resolution and temporally averaged over the 30 days preceding each fire’s recorded date. For each fire centroid, circular buffers of radius 1 km (Scale A), 5 km (Scale B), 10 km (Scale C), 20 km (Scale D), 50 km (Scale E), and 100 km (Scale F) were generated. Within each buffer, NASA-derived continuous variables (e.g., temperature, precipitation, soil moisture) were summarized using mean and standard deviation–recognizing that the 55 km grid resolution may limit representation of sub-buffer heterogeneity–while categorical predictors (e.g., land-cover type, detection method) were expressed as percent cover. Static features such as distance to roads and dominant vegetation class were extracted from the latest map layers. This multiscale, spatiotemporal aggregation ensures that each event is characterized by a comprehensive feature vector reflecting both immediate site conditions and broader environmental context.

During preprocessing, missing values are addressed, outliers are filtered, and relevant features are normalized. Meteorological variables such as temperature, humidity, wind speed, and precipitation are sourced from NASA datasets. Fire characteristics, including area burned, detection method, and suppression details, originate from KGAU reports. The Ministry’s data provides forest composition metrics, helping define dominant vegetation types that impact fire behavior. The feature engineering step includes selecting relevant factors such as meteorological conditions, vegetation type, detection method, and proximity to urban areas. The dataset is split into training and testing subsets (typically 70/30) to evaluate model performance.

Several machine learning models are applied to classify wildfires by size and risk category, each chosen based on its strengths in handling different types of data and classification tasks. XGBoost is included due to its high performance on structured data, its ability to handle missing values, and its robustness against overfitting through regularization^60^. Random Forest is used for its ensemble learning approach, which improves classification accuracy by averaging multiple decision trees and capturing complex feature interactions ^61^. K-Nearest Neighbors (KNN) serves as a non-parametric baseline model, effective for capturing local patterns in the data but requiring careful tuning to avoid sensitivity to noise ^62^. Logistic Regression is applied as a simple interpretable model that establishes baseline classification performance, particularly for distinguishing between smaller and larger fires ^63^. Decision Tree is selected for its transparency and ability to model non-linear relationships, serving as a foundation for more complex ensemble methods ^64^. The combination of these models allows for a comparative analysis, ensuring that the final classification approach balances accuracy, interpretability, and computational efficiency.

The models are evaluated using accuracy, precision, and recall, $F_1$ score. XGBoost demonstrates the highest performance and is further analyzed for feature importance using SHAP. This allows for an in-depth assessment of the contribution of individual variables, such as climatic conditions and forest composition, to fire classification.

The final pipeline can be used for applications, such as fire management, early warning systems, and resource allocation. By accurately classifying wildfires, forest management agencies can prioritize areas for prevention or mitigation measures.

## Results

### Model selection

The results of the wildfire size classification task (Table 2) show that XGBoost provides the most accurate and robust performance compared to other models. With an accuracy of 0.888, precision of 0.852, and recall of 0.888, XGBoost excels in distinguishing between fire size categories, making it the most effective model for predicting fire sizes based on the available features such as climatic data, forest composition, and detection methods.

**Table 2:** Performance metrics for wildfire classification models.

Model	Accuracy	Precision	F1-Score
XGBoost	0.888	0.852	0.862
Random Forest	0.878	0.815	0.830
K-Nearest Neighbors	0.814	0.716	0.756
Logistic Regression	0.853	0.671	0.849
Decision Tree	0.800	0.811	0.806

Random Forest performs slightly worse than XGBoost but remains competitive, likely due to its ensemble nature, which helps in handling non-linearity. K-Nearest Neighbors (KNN) and Decision Tree exhibit lower accuracy and F1-scores, indicating challenges in generalization. Logistic Regression, while offering moderate recall (0.853), has the lowest precision (0.571), suggesting difficulty in distinguishing between fire size categories.

### Classification results

**Figure 9:**
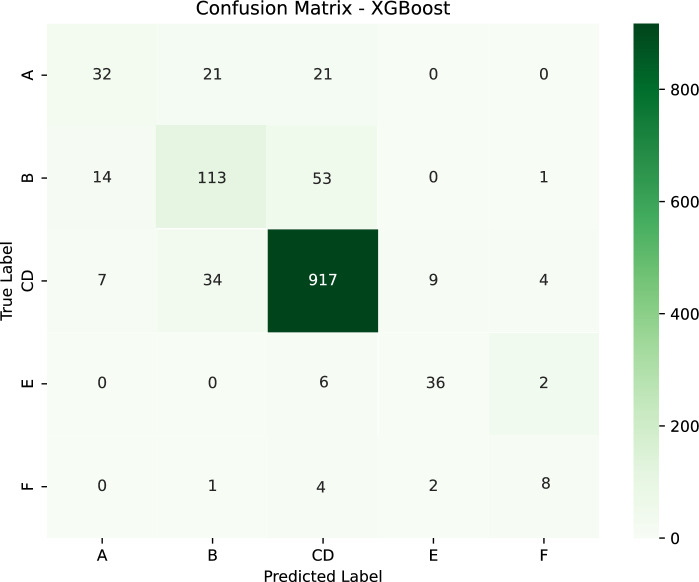
XGBoost confusion matrix.

The confusion matrix (Fig. 9) for the XGBoost model highlights key trends and challenges in predicting fire size categories.

Class A (very small fires <0.2 ha) is rarely present in the predictions, suggesting the model struggles to identify the smallest fires, likely due to limited training data or overlapping features with class B.

Class B (small fires 0.2–2 ha) is often misclassified as larger categories, particularly class E and CD. This indicates that some small fires share characteristics with larger ones, possibly due to environmental conditions or measurement limitations.

Class C and D (medium fires 2–200 ha) shows strong predictive performance, with the majority of cases correctly classified. However, some misclassifications into class E suggest that certain medium fires exhibit characteristics of larger fires, potentially due to extreme weather conditions or rapid fire spread.

Class E (large fires 201–2000 ha) is frequently confused with class C and D and F, indicating that distinguishing between large and very large fires remains challenging. This could be due to overlapping weather and terrain conditions that influence fire growth.

Class F (very large fires >2000 ha) is generally well identified, but a few instances are misclassified as class E, suggesting some fires at the lower end of the F threshold may not have distinct enough characteristics to separate them from class E fires.

Table 3 presents per-class precision, recall, and $\text {F}_1$-scores derived from the confusion matrix of the XGBoost classifier.

**Table 3:** Per-class performance metrics for XGBoost wildfire-size classification.

Class	Precision	Recall	$\text {F}_1$-Score
A (very small, <0.2 ha)	0.604	0.432	0.504
B (small, 0.2–2 ha)	0.669	0.624	0.646
CD (medium, 2–200 ha)	0.916	0.944	0.930
E (large, 201–2000 ha)	0.766	0.818	0.791
F (very large, >2000 ha)	0.533	0.533	0.533

Class A (very small fires, <0.2 ha) is rarely present in the predictions and achieves the lowest recall (0.432), suggesting that the model struggles to identify the smallest fires–likely due to limited training examples and overlapping feature values with Class B. Class B (small fires, 0.2–2 ha) attains moderate precision (0.669) and recall (0.624), but is often misclassified into larger categories (notably CD and E), indicating that some small events share environmental signatures with medium and large fires–potentially arising from similar meteorological conditions or limitations in label accuracy. Class CD (medium fires, 2–200 ha) exhibits the strongest performance (precision = 0.916, recall = 0.944, $\text {F}_1$ = 0.930), reflecting the model’s capacity to distinguish mid-sized fires. Nevertheless, misclassifications into Class E imply that extreme weather episodes or rapid fire spread can cause certain medium events to resemble large fires. Class E (large fires, 201–2000 ha) shows good but not perfect discrimination (precision = 0.766, recall = 0.818), with frequent confusion with CD and F categories; this suggests that distinguishing between large and very large fires remains challenging when their driving conditions overlap. Finally, Class F (very large fires, >2000 ha) attains a recall and precision of 0.533, indicating that while the largest fires are generally recognized, some threshold-borderline events are misclassified as Class E, likely because fires near the 2000 ha boundary lack sufficiently distinct predictive characteristics.

### Feature importance

**Figure 10:**
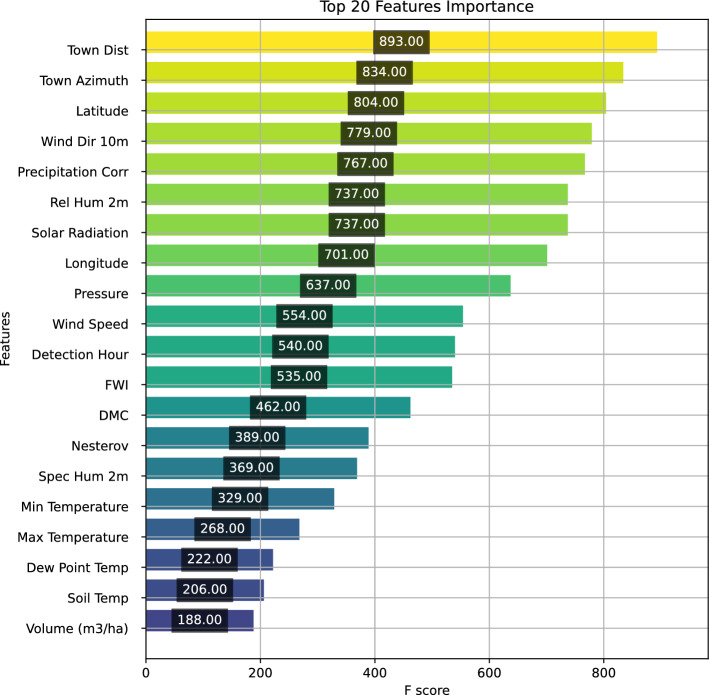
XGBoost feature scores results.

The feature score in the XGBoost model (Fig. 10) represents the number of times a given feature is used to split the data across all trees in the ensemble. A higher score indicates greater importance in influencing the model’s predictions, meaning the feature has a stronger impact on determining fire size and behavior.

It identifies town distance and town azimuth as key predictors, highlighting the impact of human proximity on fire occurrence and spread. Latitude, longitude, wind direction, and wind speed further emphasize the role of geospatial and meteorological factors, with wind being a major driver of fire movement. Precipitation correlation, solar radiation, and relative humidity indicate that dry conditions and high solar exposure increase fire risk. Fire weather index and duff moisture code reinforce the importance of fuel dryness in fire escalation. Detection hour suggests that early detection can enhance control efforts ^65^. Pressure, Nesterov index, and specific humidity provide additional weather-related insights, affecting fuel moisture and fire intensity. Minimum and maximum temperature, soil temperature, and dew point temperature contribute but have a relatively lower impact. Vegetation volume is less critical than atmospheric and human factors. These findings underscore the need for real-time meteorological monitoring, early warning systems, controlled burns, and urban fire prevention policies to mitigate fire risks effectively.

### SHAP analysis

**Figure 11:**
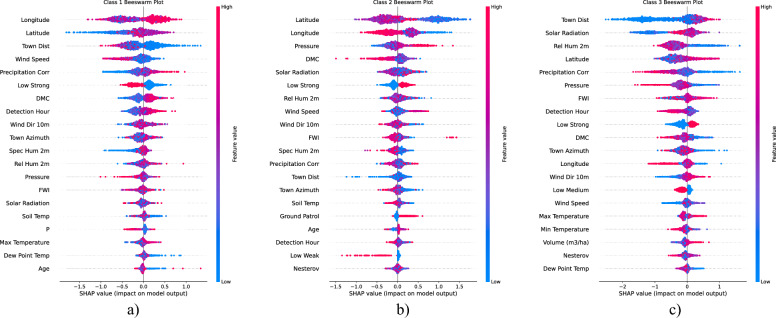
SHAP beeswarm plots for: (**a**) Class B (small fires) (**b**) Classes C and D combined (medium fires) (**c**) Class E (large fire).

SHAP beeswarm plots in Fig. 11 illustrate the impact of different features on the model’s predictions for fire size classification. It highlights the progressive role of environmental and climatic factors as fires increase in size. While small fires are largely influenced by geospatial and short-term meteorological conditions, larger fires are more dependent on fuel dryness, fire weather indices, and long-term climate patterns

It should be noted that SHAP values quantify each feature’s contribution to the XGBoost model’s output rather than establishing mechanistic causality in wildfire processes ^66^. In other words, SHAP decomposes the prediction into additive contributions of individual predictors, reflecting how the model internally attributes importance under the given training data distribution ^67^. Consequently, high SHAP values for a variable (e.g., extended dry periods) indicate its strong influence on the model’s classification decision, but do not by themselves demonstrate that this factor is a direct or sole driver of fire size in the real world. However, high SHAP value does not imply causation but reflects model influence within the current dataset.

Small fires are predominantly influenced by geospatial factors, indicating that certain locations may have a higher likelihood of small fire occurrences ^68^. Proximity to towns (Town Distance) suggests human-related ignition sources, possibly linked to recreational activities, power lines, or small-scale land clearing. Wind speed and precipitation correlation suggesting that small fires are sensitive to immediate atmospheric conditions. Higher wind speeds may facilitate fire spread, whereas precipitation can help suppress ignition. The detection hour is also a contributing factor, which could indicate that human observation patterns (e.g., more active fire monitoring during daylight hours) affect the classification of small fires. Early detection and rapid response strategies in high-risk locations can prevent small fires from escalating. Targeted awareness campaigns in areas close to human settlements may help mitigate anthropogenic fire ignitions. Wind monitoring can help predict small fire risks and inform suppression efforts.

Medium fires exhibit a broader set of influential factors compared to small fires. Fire weather conditions and fuel moisture content (Duff Moisture Code, DMC; Fire Weather Index, FWI; Pressure) are dominant in fire spread within this size range. The higher importance of solar radiation and humidity suggests that medium fires are more strongly influenced by atmospheric stability and evaporation rates, leading to drier fuel conditions. Wind speed remains a key factor, highlighting its role in fire expansion beyond the small fire category ^69^. The combination of fire weather indices and meteorological variables indicates that medium fires require both dry conditions and sufficient wind energy to sustain growth. Interestingly, town distance remains a key factor, which may imply that some medium fires originate from human activities but grow larger due to favorable environmental conditions ^70^. Fire danger prediction models should prioritize fire weather indices (FWI, DMC) to identify areas at risk for medium-sized fires. Fuel management strategies, such as prescribed burns and vegetation clearance, can reduce available combustible material. Infrastructure protection measures should be enhanced in areas where human presence coincides with fire-prone conditions.

Large fires exhibit a complex interplay between geospatial, meteorological, and fire weather variables. Unlike smaller fire categories, large fires are more heavily influenced by long-term climatic conditions rather than immediate weather fluctuations. The dominance of town distance suggests that while some large fires originate in remote areas, their spread may still be linked to human activity, infrastructure, or land use practices. Precipitation correlation and pressure indicate that long-term dry conditions and atmospheric stability impact fire growth ^71^. FWI and DMC remain key factors, emphasizing that fuel availability and dryness are the most contributors to extreme fire spread. Additionally, soil temperature becomes an important factor, suggesting that surface heat accumulation may contribute to sustained burning conditions. Climate-driven fire risk assessment is necessary, as large fires are influenced by long-term weather patterns rather than daily fluctuations. Strategic firebreaks and landscape-level fuel reduction can help contain fires before they escalate to extreme sizes ^72^. Resource allocation should focus on preemptive suppression strategies in regions where fire-prone conditions persist over longer periods.

## Discussion

The forests of Krasnoyarsk Krai vary in structure and composition depending on latitude, elevation, and local climatic conditions. In the north, larch-dominated stands transition into sparse forest-tundra landscapes, while central and southern areas contain denser coniferous and mixed forests. These forests are shaped by disturbances, with wildfires being one of the most recurrent factors influencing forest dynamics. Fire regimes in the region are influenced by seasonal weather patterns, fuel availability, and ignition sources, both natural and human-caused ^73^. The data indicate that years with higher fire activity coincide with prolonged dry conditions, elevated temperatures, and increased atmospheric instability, which contribute to lightning-induced ignitions. Years with fewer fires suggest more stable moisture levels and possibly improved suppression efforts.

The interaction between forest composition and fire occurrence in the region requires further study. Coniferous forests, where dominated by pine and spruce, have higher flammability due to the resinous nature of their foliage and accumulated litter ^74^. Deciduous forests, such as those with birch and aspen, are often less susceptible to fire spread due to higher moisture content in their leaves and lower accumulation of dry, combustible material. However, changes in species distribution over time, influenced by fire frequency and post-fire regeneration patterns, may alter overall fire susceptibility. Understanding these shifts is necessary for predicting long-term fire behavior.

Although the XGBoost model has shown strong performance in classifying wildfire sizes based on climatic and environmental features, the predictive capacity of any model is constrained by the quality and resolution of the input data. Climatic variables are typically averaged over broad spatial scales, which may not capture local microclimatic effects that influence fire ignition and spread ^75,76^. Similarly, variations in fuel structure, topography, and wind conditions are not always well represented in fire occurrence datasets ^77^. Future work could incorporate finer-scale environmental variables, such as real-time soil moisture and wind speed data, to refine fire size predictions ^78^.

Detection-related uncertainty is an inherent aspect of wildfire datasets compiled from multiple sources, including satellite observations, aerial surveys, and ground patrols. Each detection method has varying spatial and temporal resolution, sensitivity thresholds, and coverage consistency, which can influence the accuracy of recorded fire perimeters and, consequently, the estimated fire size. For example, small and short-lived surface fires may go undetected by satellites due to cloud cover, coarse resolution, or infrequent overpasses, while ground-based observations may underreport remote or inaccessible events. These disparities can introduce biases in the class distribution, particularly underrepresenting very small fires (Class A) and overestimating the extent of merged fire complexes. Acknowledging such uncertainties is essential when interpreting model performance and generalizability, as they may affect both the training process and evaluation of predictive accuracy across fire size categorie

Several constraints inherent to the present framework merit explicit acknowledgment. First, the employed meteorological inputs derive primarily from NASA reanalysis products, which provide grid-based data at resolutions between $0.25^\circ$ and $2.5^\circ$ ^79^. Such coarse granularity fails to represent microclimatic heterogeneity–such as cold-air pooling in topographic depressions, canopy-level moisture gradients, and small-scale wind eddies–that critically influence fire ignition and early propagation. Consequently, feature-importance estimates and model fidelity may be biased in sectors where sub-grid processes dominate risk.

Second, very small fires (Class A; < 0.1 ha) are substantially underrepresented due to satellite detection thresholds and uneven ground-patrol reporting ^80^. This class imbalance can skew model training toward medium and large fires, reducing sensitivity to small events and inflating overall performance metrics when minor fires are misclassified or omitted. Implementation of synthetic minority oversampling or targeted field surveys could ameliorate this imbalance and improve classifier robustness across all size classes ^81^.

Third, key biophysical and ignition-source variables remain excluded from the predictor suite. Lightning frequency–recognized as the primary natural ignition mechanism in boreal ecosystems–is absent, limiting discrimination between human- and lightning-initiated fires ^82,83^. Soil texture and moisture, which govern ignition susceptibility and fire spread dynamics, are also omitted, as are quantitative measures of fuel load (e.g., litter depth, duff moisture content) and vegetation structure (e.g., crown bulk density from LiDAR). Integration of spatially explicit lightning strike datasets, in situ fuel-moisture observations, and high-resolution vegetation structure metrics is expected to enhance both predictive accuracy and ecological interpretability ^84^.

By characterizing these limitations, the framework is positioned as an exploratory, region-specific proof of concept. Future extensions should prioritize (1) incorporation of finer-scale meteorological and ignition-source data ^85^, (2) balanced representation of all fire-size categories through data augmentation or enhanced detection protocols ^86,87^, and (3) inclusion of detailed soil and fuel variables ^88^.

Another limitation lies in the temporal scope of the analysis. While the dataset spans 2010 to 2024, fire regimes in boreal and subarctic forests operate over much longer timescales, often shaped by decadal or even century-long climatic oscillations. Examining historical fire records alongside paleoclimate reconstructions could help contextualize the observed trends within broader climatic cycles ^89,90^. Additionally, assessing how human-driven landscape changes–such as logging, road construction, and industrial activity–modify fire behavior would provide a more comprehensive understanding of fire risk in the region ^91^.

Further refinement of fire prediction models could also benefit from incorporating post-fire recovery data ^92^. Vegetation regrowth, soil erosion, and carbon fluxes following fire events influence future fire susceptibility. Remote sensing data, combined with long-term field studies, could improve assessments of how different forest types recover from fire and whether certain areas transition toward more fire-prone ecosystems over time.

Addressing these gaps would improve the ability to forecast wildfire risks under varying climatic conditions and inform land management strategies aimed at reducing fire hazards while maintaining the ecological functions of Krasnoyarsk Krai’s forests.

## Conclusions

The XGBoost classifier achieved the highest accuracy (0.888) and balanced precision–recall compared to Random Forest, KNN, Logistic Regression, and Decision Tree, demonstrating its ability to distinguish fire sizes using climatic data, forest composition, and detection methods. However, the confusion matrix highlights underperformance on very small fires–likely due to scarce training examples–and occasional misclassification between adjacent size classes, reflecting gradual changes in fuel and forest structure. SHAP-based feature analysis identifies proximity to human settlements, geospatial coordinates, and wind metrics as key predictors, while meteorological factors (precipitation, solar radiation, relative humidity, FWI, DMC) govern fuel dryness and fire spread. Small fires correlate with short-term weather and location effects, whereas medium and large fires depend on sustained dry conditions and fuel deficits.

These findings can inform refined forest management in Krasnoyarsk Krai. Incorporating high-resolution remote sensing and localized weather data will enhance the spatial and temporal accuracy of fire risk maps, enabling more precise fuel treatments (e.g., controlled burns, vegetation clearance) and optimized surveillance and resource allocation. Future efforts should expand the dataset to capture underrepresented fire classes and integrate post-fire feedback, allowing the model to evolve with changing forest conditions and support nuanced fire management across the region. While the model demonstrates strong performance within the study region, its application to other areas should be preceded by local calibration to account for region-specific conditions

## Data Availability

The code and datasets for this research are openly accessible on the Git repository SibFires.
